# Trigeminal electrical stimulation with ULFTENS of the dorsal anterior mucosal surface of the tongue: Effects on Heart Rate Variability (HRV)

**DOI:** 10.1371/journal.pone.0285464

**Published:** 2023-05-10

**Authors:** A. Monaco, R. Cattaneo, P. Smurra, S. Di Nicolantonio, F. Cipriano, D. Pietropaoli, E. Ortu

**Affiliations:** Departement of Clinical Medicine, Public Health, Life and Environmental Sciences, University of L’Aquila, L’Aquila, Italy; BG-Universitatsklinikum Bergmannsheil, Ruhr-Universitat Bochum, GERMANY

## Abstract

**Background:**

Trigeminal electrical stimulation of the dorsal anterior mucosal surface of the tongue has demonstrated its efficacy in a variety of neurological disorders in which anatomical or functional alterations are present. The pathogenesis of such disorders is often linked to altered arousal circuits, and the benefits of tongue stimulation are attributed to the rebalancing of this system. Dental ULFTENS shows efficacy in acting on the muscular, autonomic system and control of the descending pathways that modulate pain. It is administered at the skin level in the area anterior to the tragus and not on the mucosal surface of the tongue. The use of this stimulation technique at the tongue level could have new applications and clinical results if it were able to reduce the activity of arousal circuits.

**Material and method:**

A new intraoral device allowed electrical stimulation of the dorsal anterior mucosa of the tongue in 32 healthy young women. The effects on HRV were monitored by photoplethysmographic wave (PPG) and compared with a control group. The HRV parameters studied were RMSSD, HF, LF, LF/HF, REC, DET.

**Results:**

The group of stimulated subjects showed a significant change in some of the HRV parameters that was maintained even in the epoch after the end of electrical stimulation. This effect can be considered as a vagal activation and a change of HRV trend. The control group of unstimulated subjects showed an opposite trend. There were no undesirable or annoying effects of stimulation.

**Conclusion:**

Stimulation of the dorsal anterior (trigeminal) mucosal surface of the tongue with ULFTENS applied with an intraoral device was shown to be able to increase HRV.

## Introduction

Trigeminal sensory in recent years has been shown to be associated with the functioning and dysfunction of the central nervous system, and it has been suggested that its alteration may play a role in the genesis or, at least, in the worsening of chronic neurodegenerative disorders [1, 2].

Probably this action is partly related to the relationship between the trigeminal and arousal systems and, in particular, to the activity of the locus coeruleus [1, 3, 4].

Transcutaneous trigeminal stimulation together with vagus stimulation has been used in recent years to treat a number of neurological disorders on the basis of a mechanism of action that is not well elucidated but may involve trigeminal vagal connections with arousal circuits [5–8].

Dental ULFTENS (Ultra-Low Frequency Transcutaneous Electrical Nerve Stimulation) is a transcutaneous trigeminal electrical stimulation technique that has long been used in dentistry for TMD (temporo-mandibular disorder) diagnosis and treatment, orthodontic diagnosis and treatment, and to facilitate prosthetic rehabilitation [9–11].

This technique uses a stimulation frequency of 0.66 hz and is applied bilaterally at the level of the skin area anterior to the tragus. Apart from its effect on myoelectric tone [12], it has been suggested that its mechanism of action may in part be mediated by the autonomic system and descending pathways on the control of sensory afferents [13].

At the same time, superficial electrical stimulation of the dorsal anterior surface of the tongue mucosa has been used to promote functional sensory and motor recovery of syndromes caused by anatomical damage to the central nervous system [14], and it has been suggested that trigeminal stimulation of the dorsal anterior surface of the tongue mucosa may be a privileged and powerful channel for communication with the central nervous system (Tongue-Brain machine) [15]. The mechanism of action is not completely clear, but it could also in part be related to an action at the level of the brainstem proper of the structures that preside over the control of arousal [16].

One of the main effects of the action of the brainstem noradrenergic systems is related to cardiac activity by controlling the balance between sympathetic and parasympathetic vagal activity [17, 18]. Vagal electrical stimulation has shown effects on both arousal [19] and cardiac activity [20, 21]. While some work shows that vagal stimulation induces a response not only at the locus coeruleus but also at the trigeminal level [22].

One of the methods to assess the activity of the arousal system is related to the possibility of monitoring cardiac activity through some parameters of HRV (heart rate variability) [23, 24]. The possibility of assessing through HRV the effects of trigeminal stimulation of dental ULFTENS has already been discussed elsewhere [13].

Our study attempts to combine stimulation of the dorsal anterior surface of the tongue with the dental ULFTENS type of electrical stimulation on the hypothesis that such stimulation has a significant effect on the arousal centers called upon for both stomatognathic system physiology and TMDs [25].

Such stimulation could be useful for the dental clinic, but it has, at present, never been performed and, for that reason, it is not known whether such stimulation could have a central action. Therefore, we constructed an ad hoc device to stimulate the dorsal anterior surface of the tongue with dental ULFTENS and evaluated the effect on cardiac activity by BVP (Blood Volume Pulse) obtained from the PPG wave (Photoplethysmographic wave).

## Materials and method

### Composition of the groups

This study was carried out in accordance with the fundamental principles of the Declaration of Helsinki and was approved by the Internal Review Board (IRB) of the University of L’Aquila (Number 16137/2016).

Written informed consent was obtained from all the participants.

For the composition of the 2 working groups, 64 healthy young women were recruited from among female students in the undergraduate programs of Dentistry, Hygiene, and Medicine at the University of L’Aquila. Inclusion criteria were: good health status and physical fitness, female sex, age between 20 and 30 years (Control group: mean age 23,65 ds 5,39,; case group: mean age 24,53, sd 2,79; p = 0.32), non-smoker, no more than 3 espresso coffees per day, no intake of stimulant or psychotropic drugs in general, body mass index between 18.5 and 24.9. Exclusion criteria included the presence of acute and/or chronic cardiocirculatory and respiratory disorders, metabolic and autonomic system disorders, intake of peripheral and central nervous system excitatory or inhibiting substances, presence of generalized anxiety and/or panic attacks, or mood disorders.

Women who presented with menstrual phase at the time of registration were referred to a period outside this cycle phase.

The two groups were created randomly by assigning the person to one group or the other based on a coin toss. Recordings were made between 10 am and 12 am over a period of 3 months.

Before each recording session, people were warned to eat a light breakfast and not to consume any exciting substances such as coffee or caffeinated beverages. They were also asked to empty their bladders before the instrumental recording began.

The candidates were advised of the length of the recording session, but they were not aware of what would happen during the session. At the beginning of the recording the control group members were told that they would have to remain still with their eyes closed for about 20 minutes during which a pulse oximeter would record their heart activity. Those belonging to the case group were shown the tongue stimulator and told what would happen during the session itself and that during all this time a pulse oximeter would record heart rate.

For this second group, the instructions were as follows, "You will hold the pacifier (pacifier) in your mouth, the whole time of the session. At some point you will feel a slight pinch on the front of your tongue. That is the sign of the beginning of electrical stimulation of the mucosa of the tongue. At some point the stimulation will be stopped and, as a result, you will no longer feel any stimulation on the tongue. You will still have to remain with your eyes closed and still for a few minutes before the recording session is over."

None of the participants in this study had any problems completing the recording session, and none of those in the case group complained of discomfort or other reasons for stopping the recording itself. No side effects were observed during the recording sessions.

### Instrumentation

#### PPG wave for HRV

HRV analysis was conducted on the photoplethysmographic signal. The PPG wave was collected with the ProComp Infiniti instrument (Thought Technology 5250 Ferrier St, Suite 812, Montreal, Quebec H4P 1L3). The PPG wave was acquired by sampling at 2048 data per second in order to obtain a detailed curve that would allow the precise measurement of heart rate. The PPG signal was processed by the included software to provide the IBI (interbeat interval). The sequence of the various IBIs was transformed into txt and further processed through the Kubios software. The following parameters were selected from the Kubios suite: RMSSD (Root mean sqare deviation standard) for time domain; LF (Low frequency), HF (High Frequency), LF/HF for frequency domain; REC (Recurrence) and DET (Determinism) for nonlinear analysis.

A respiratory amplitude and frequency signal was, in addition, recorded with a specific voltage sensor simultaneously with the PPG signal.

#### ULFTENS

ULFTENS for dental use has been used for decades in dentistry and takes advantage of ultra-low-frequency (0.66 hz) stimulation, characterized by a square wave with active phase lasting 500 microsec, with an amplitude adjustable from 0 to 24 milliamperes. It features a reference electrode to be placed on the nape of the neck and an active electrode split into two channels (right and left) that allow left and right synchronous stimulation. Traditionally, the skin stimulation area is at the front of the left and right tragus. The instrument, dental procedure, and stimulation characteristics have been extensively described elsewhere [11, 26].

In the case of our study, the nuchal reference electrode was retained, while the two stimulation channels on the right and left were connected to two electrodes embedded in the intraoral device through two leads coming out separately from the front of the device.

This made it possible to administer ULFTENS on the dorsal anterior surface of the mucosa of the tongue. The amplitude of stimulus delivery was subjective and determined by the study subject’s perception of the stimulus. On average, the stimulus was felt around 1.5–2.5 milliamperes about 50 percent lower than the amplitude required to reach the sensory threshold for trigeminal stimulation at the level of the area in front of the tragus.

#### Intraoral device for electrical stimulation of the tongue

**Fig 1:**
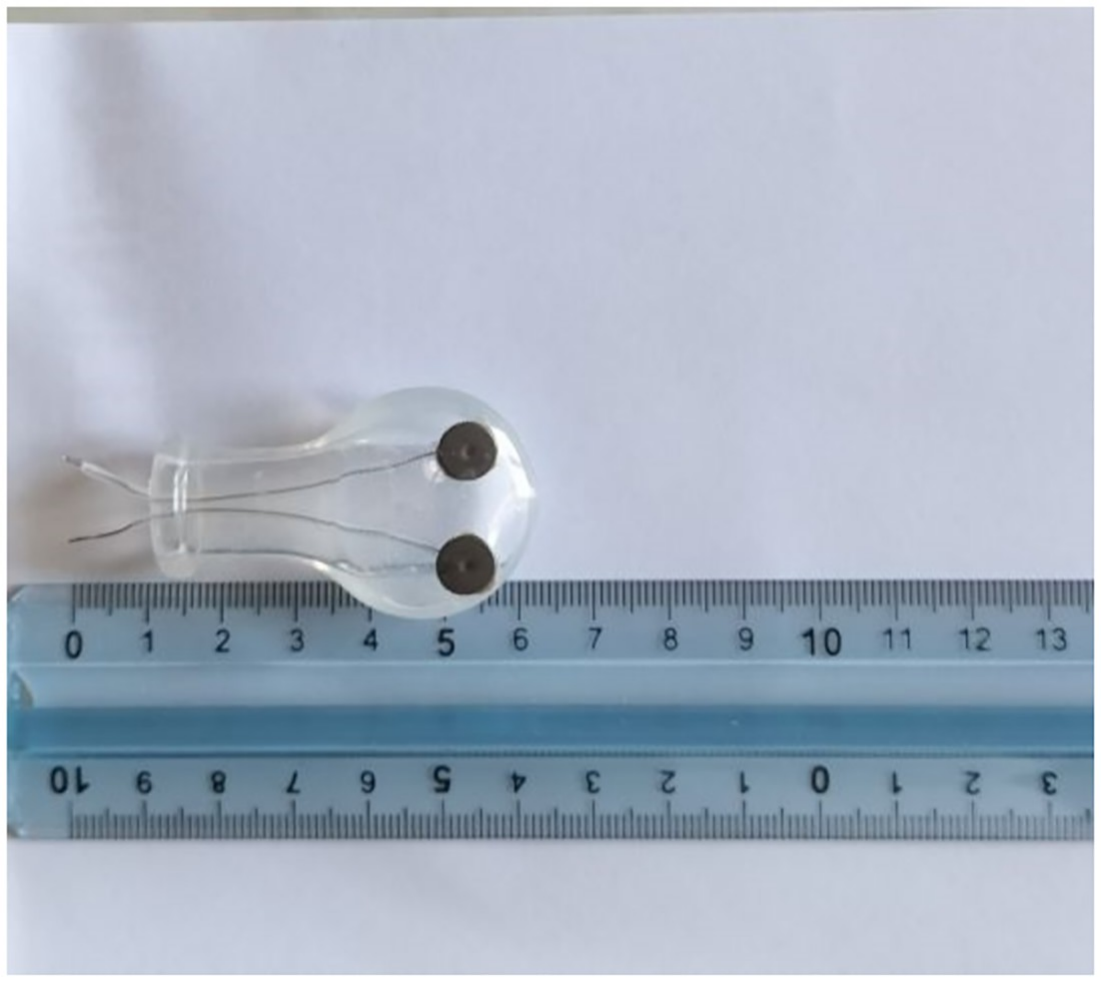
Pacifier with applied electrodes for electrical stimulation of the dorsal anterior surface of the tongue.

Fig 1 shows the example of an intraoral device used for ULFTENS of the mucosal surface of the tongue. It is constructed on the basis of a product marketed on the web (LittleforBig.com) that is empty inside and open in its "handle" part; it has been modified ad hoc for our purpose. The stimulating electrodes are made from duotrode electromyography electrodes from Myotronics (5870 S. 194th Street Kent, WA 98032–2125); these electrodes are inserted into the ventral surface of the pacifier and connected to the outside through a cable of conducting material covered with an insulating silicone tube used in orthodontics, all of which is contained within the empty space of the pacifier. The tapered part of the pacifier, the "handle," is also open, and out of it are run the leads that will be connected to the electrostimulator through "alligator" connectors. The right electrode is connected with the right lead of the electrostimulator and the left electrode is connected with the corresponding left lead of the electrostimulator. The person holds this instrument in the mouth exactly as he or she would hold any pacifier without experiencing difficulty or fatigue. The "handle” part comes out of the lips and the connection with the electrostimulator is made outside the mouth. No subject in the course of this study had any problems with such a device.

#### Recording protocol

The environment was maintained at constant temperature and humidity. Light was soft and constant, no noisy disturbance was present during the recording. The person was made to lie on a medical examination couch, and a small, soft pillow was provided to those who felt they were more comfortable in this way. The methods of registration as referred to above were explained. After a few minutes (usually 5–6) of acclimatization, the PPG sensor was applied to the fingertip of the middle finger of the right hand.

People who would receive ULFTENS were given the pacifier, explained what it was and what would happen during electrical stimulation of the mucosal surface of the tongue. The "ground" electrode was applied to the nape of the neck and connected with the electrostimulator. The pacifier was placed in the mouth and it was also connected with the electrostimulator turned off, so that at the time of stimulation prolonged manipulation of the person and instruments was not necessary.

At this point the recording session would start. People were asked to sit still with their eyes closed for the entire recording. The tracings were checked online by a researcher unaware of the reason for the recording for errors in the PPG sensor application procedure, and if necessary, appropriate corrections were made at this stage. The recording traces were followed online by the same researcher who was unaware of the reason and procedure for recording. A second researcher was delegated to deliver the pacifier according to the randomization of the groups and the stimulation procedure for the subjects belonging to the case group. Six minutes considered as baseline were acquired T0 (Time 0) for the control group and Before ULFTENS for the case group, then next 6 minutes considered as T1 (Time 1) for the control group and ULFTENS for the case group. Finally, another 6 minutes were acquired T2 (Time 2) and After ULFTENS for the two groups respectively. The three epochs were acquired continuously without interruption between them.

In the case group, the procedure involving turning on and reaching the perceptible stimulus on the tongue never exceeded 15 seconds. This period was not edited accepting the possible "polluting" effect of such 15 seconds on the electrical stimulation epoch data.

Blood Volume Pulse traces obtained with PPG were visually checked online for signal alterations due to incorrect procedure or software connection problems. Once the signals were accepted and recording started, no trials needed intervention or interruption.

**Fig 2:**
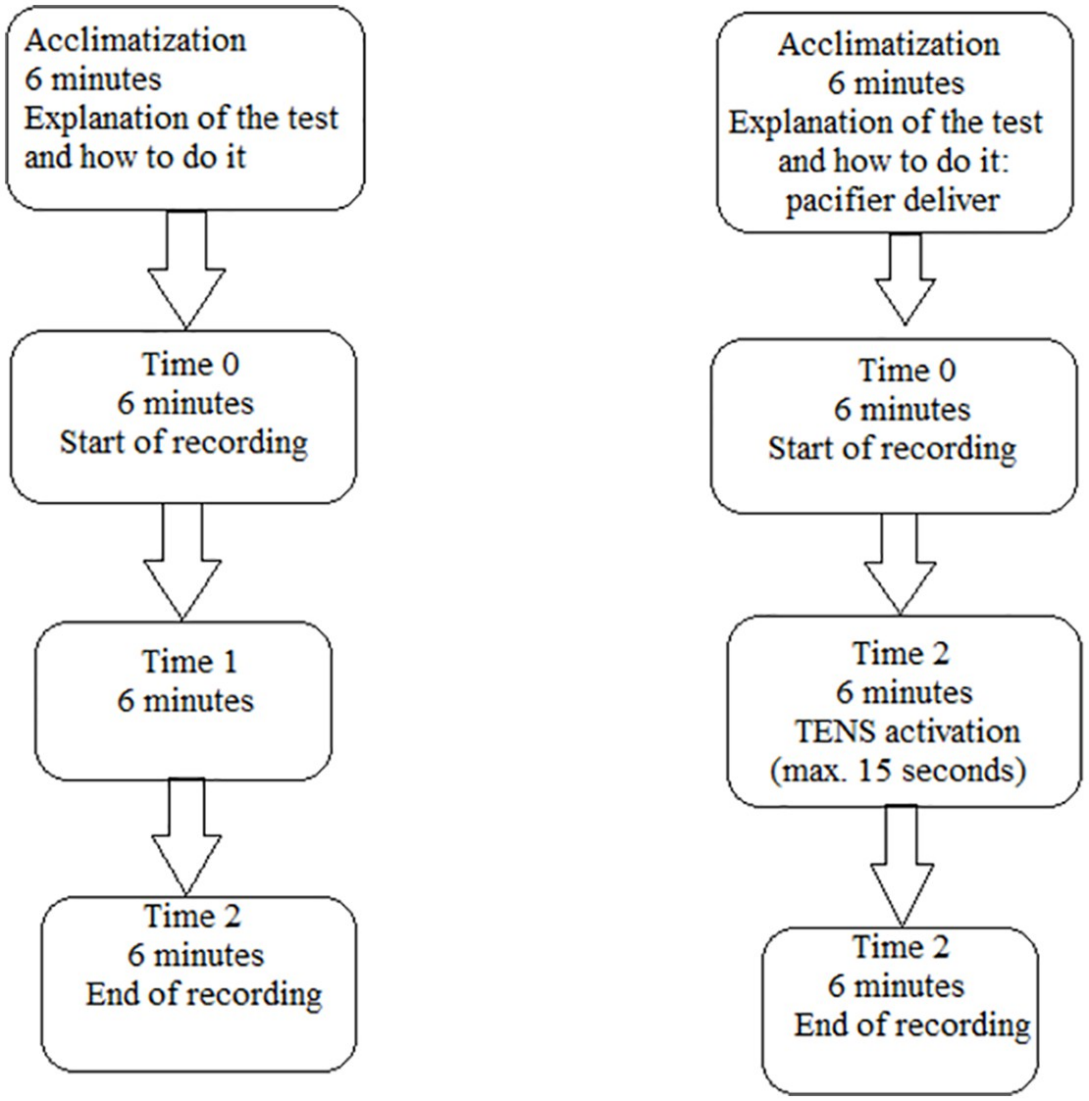
Timeline of the recording session.

Fig 2 shows the timeline of the recording session and the differences between cases group and control group.

**Fig 3:**
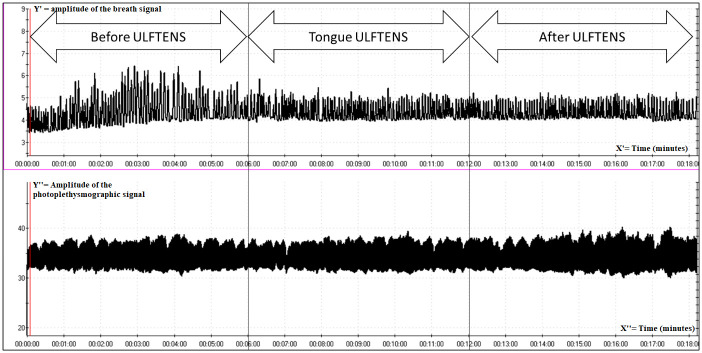
Tracing of PPG recording (lower part) and abdominal breath (upper part) of a subject in the study group. At minute 6, lower time bar (first vertical line), ULFTENS starts, at minute 12 (second vertical line) ULFTENS is turned off. The X-axes (X’ and X”) represent time in minutes. The Y’-axis represents the amplitude of breath signal while Y”-axis represents the amplitude of photopletismographic signal.

Fig 3 shows a complete PPG recording of a person who received lingual ULFTENS.

**Fig 4:**
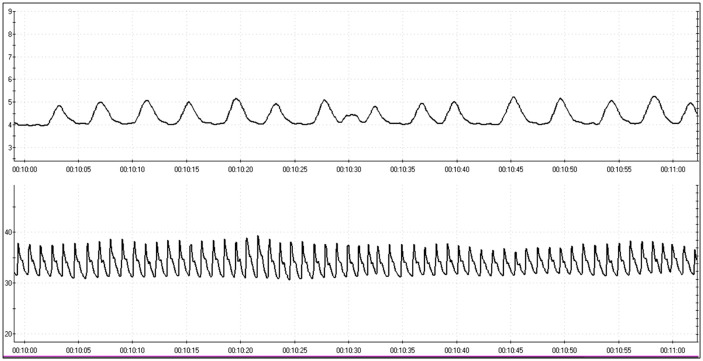
About one minute of recording (from minute 10 to minute 11 (see the lower time bar)).

Fig 4 shows one minute of the recording of Fig 3 so as to appreciate the shape of the raw wave recorded by the system.

**Fig 5:**
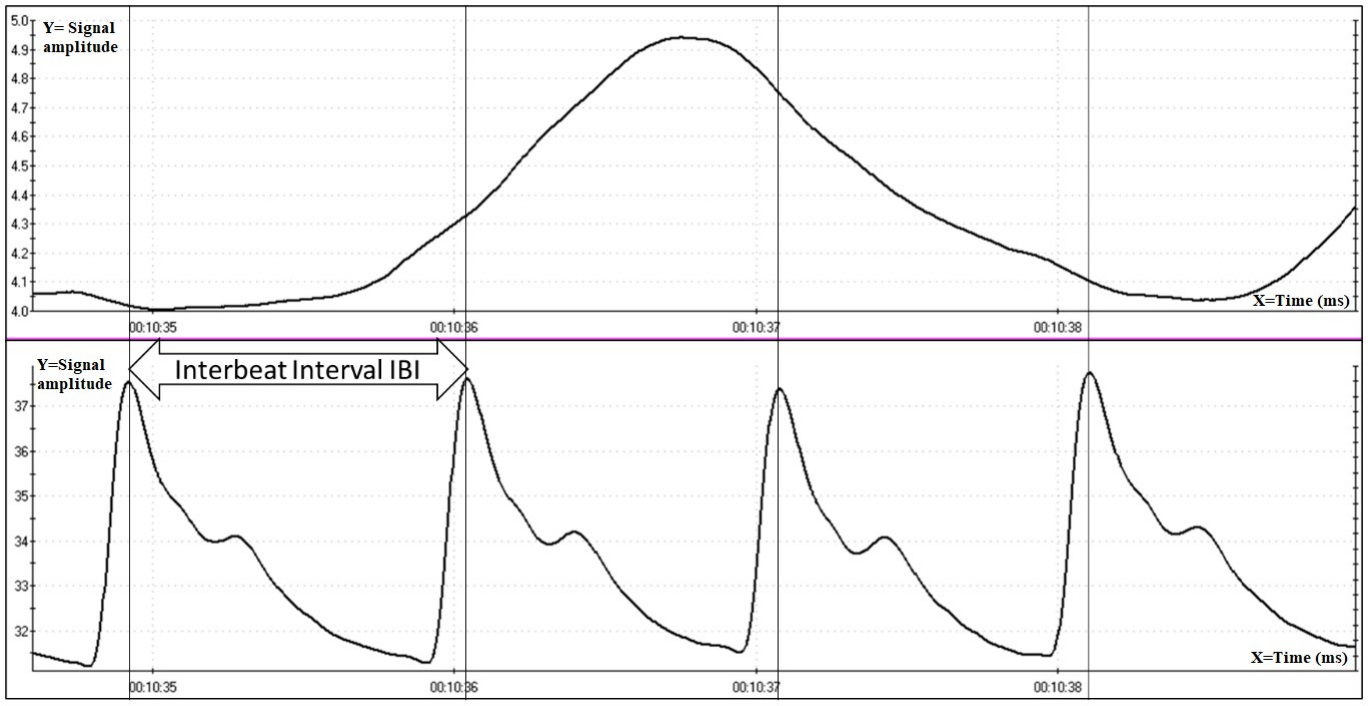
About 7 seconds of recording (from minute 10.24 to minute 10.31) in which there are 4 PPG waves and one abdominal breath wave. The vertical lines were drawn at the point of maximum amplitude of the PPG wave. The interbeat interval (IBI) is the measurement in milliseconds between the apexes of two consecutive PPG waves. The X-axis represents time in milliseconds while Y-axis represents the signal amplitude.

Fig 5 shows the magnification of 4 PPG waves sampled at 2048 hz for automatic IBI analysis. The vertical lines are drawn at the point of maximum amplitude of the PPG waves corresponding to the maximum amplitude of the Blood Volume Pulse.

#### Statistics

Statistical analysis was performed by a researcher unaware of the procedures and rationale of the study. Wilcoxon signed-rank test was adopted to verify any difference between T0, T1 and T2 in terms of investigated variables (RMSSD, LF, HF, LF/HF, REC, DET). Bonferroni correction was applied as appropriate. Data visualization was implemented using values z-scores.

The significance of the p statistic was set at 0.05.

## Results

**Fig 6:**
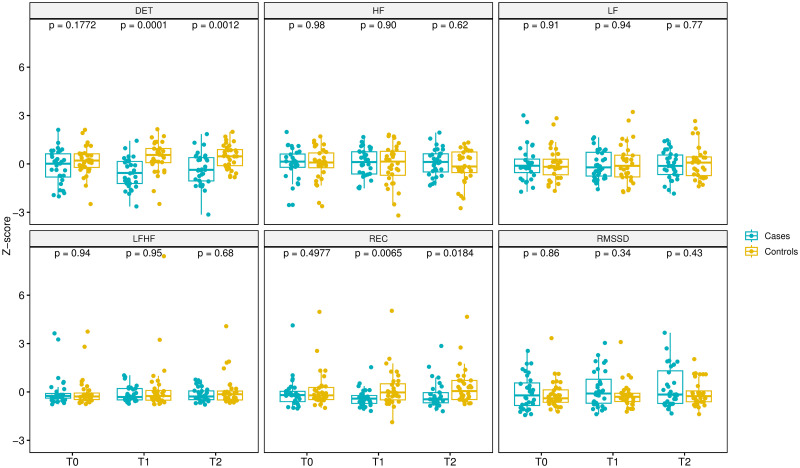
Wilcoxon unadjusted test. Comparison of parameters between cases (light blue) and controls (yellow).

Fig 6 compares each cases parameter with that of the controls at T0, T1 and T2 according to an unadjusted Wilcoxon test. For each HRV parameters (RMSSD, LF, HF, LF/HF, DET and REC) the p-value was calculated by comparing cases and control in T0 vs T1, T0 vs T2, T1 vs T2. In the baseline condition (T0) none of the parameters considered is statistically significant. This allows the two groups to be considered comparable. Focusing on the parameters DET and REC, at case-control comparison there is statistical significance for both at T1 (DET, p = 0.0001; REC, p = 0.0065). This phenomenon also recurs at T2 (DET, p = 0.0012; REC, p = 0.0184). This means that these two parameters in the cases have a different trend at T1 than in the controls, which is also maintained when stimulation is stopped (T2).

Evaluating HF, LF, and LF/HF, at both T1 and T2 no comparison of HRV parameters between the controls and cases groups show a statistically significant change.

In conclusion, when comparing the cases with the controls, not all HRV parameters change at the time of lingual stimulation (T1) compared to the basal condition, only the parameters of the non-linear analysis, DET and REC, show a significant alteration both in the comparison at T1 and in the comparison at T2.

One could therefore assume in a partial alteration of the HRV trend with regard to the parameters DET and REC following tongue stimulation with ultra-low-frequency ULFTENS.

## Discussion

Our work highlighted the following points:

1. Trigeminal electrical stimulation of the dorsal anterior mucosal surface of the tongue with dental ULFTENS through a special intraoral device is feasible and gave no adverse or side effects in healthy young women;
2. HRV obtained with PPG and measured through the parameters RMSSD, LF, HF, LF/HF, REC, and DET significantly change with 6-min duration stimulation for two parameters (DET and REC) and remained significantly so in the six minutes following stimulation when comparing cases and controls;

The results of our work indicate that stimulation of the dorsal anterior surface of the mucous membrane of the tongue influences certain HRV parameters (DET and REC) by comparing the group of cases and controls at the time of tongue stimulation (T1) and immediately after its discontinuation (T2).

In our study, we detected HRV parameters using a PPG signal from the middle finger of the right hand. The PPG wave is considered a good surrogate for the ECG (electrocardiogram) with regard to the detection of HRV parameters in both healthy subjects [27] and subjects with cardiac disorders [28], especially if the detection is performed in the supine position [29]. Consequently, the data collected in our study can be considered congruous for the purpose of this work.

The existence of a close anatomo-physiological relationship between the trigeminal and vagal systems is demonstrated by the presence of trigeminal cardiac reflexes predicting bradycardia to asystole upon peripheral trigeminal stimulation. These reflexes can be triggered by any trigeminal afferent branch and indicate the existence of a neuroanatomical reflex pathway between a trigeminal sensory afferent arm and a vagal efferent arm that induces the change in cardiac activity [30, 31].

Although the trigeminal cardiac reflex exists, attesting to the existence of a mono- or paucisynaptic circuit between the trigeminal afferent periphery and the vagal motor response directed to the heart, is it possible the existence of a more diffuse circuit for the physiological and functional integration between the two sensory and motor systems of the trigeminal and vagus?

Spinal, trigeminal, and vagal sensory afferents have long been known to project at the level of the trigeminal sensory nuclei to an area, included anatomically within the nuclei themselves, called the paratrigeminal nucleus. This area, specifically, is intercalated on the vagal cardiorespiratory pathways [32]. At the same time, an extremely dense network of trigeminal endings has been described homolaterally in the paratrigeminal nucleus and the nucleus of the solitary tract [33]. In addition, vagal afferent pathways directed to the paratrigeminal nucleus also directly project monosynaptically to the spinal nucleus and the main sensory nucleus of the trigeminal nucleus [34]. Interestingly, recently (2019) transauricular electrical stimulation of the vagus itself has been reviewed, partially, attributing part of the "vagal" effect to stimulation of trigeminal afferents and direct central trigeminal connections that would convey trigeminal signals from the periphery to the sensory and motor nuclei of the vagus and the nuclei of the arousal system (Locus Coeruleus) [35].

The "vagal" afferents, according to Henssen (2019) [36], would project to 4 trigeminal nuclei, one of which would be represented by the Trigeminal Spinal Nucleus, which thus should be considered part of the vagal nuclear circuit. Vagal sensory afferent fibers would intersect the trigeminal spinal tract and terminate directly at the level of the trigeminal spinal nucleus suggesting the existence of a trigeminal vagal anatomic complex [37]. Vagus stimulation exerts an influence on arousal centers; this has been suggested through the change in pupil size and reduction in cortical alpha rhythm [19] following peripheral vagus stimulation. Such central influence seems, moreover, to induce an enhancement, in a broad sense, of cortical activities [38, 39].

Although Data in the literature are mixed regarding the finding of a change in pupil size as a central effect of electrical stimulation of the vagus [40], peripheral cardiovascular effects are less uncertain [41]. In a general sense, however, the literature seems to favor that vagus stimulation can induce modulation of arousal centers [42]. In this sense, probably, the effects on cardiac activity would consist mainly of an inhibition of sympathetic activity rather than direct parasympathetic activation [43]. It would be, therefore, the inhibitory vagal action on sympathetic activity that Porges (2022) calls the "vagus brake" [23].

It seems possible that transauricular vagal stimulation of the vagus is capable of activating in addition to the cardioregulatory vagal nuclei also the noradrenergic truncated encephalic structures of the locus coeruleus and the serotonergic structures of the raphe [44]. On the other hand, transcutaneous trigeminal electrical stimulation also appears to involve, various central effects: modulating cortical activity in both healthy subjects and those with drug-resistant epilepsy [45, 46] probably by increasing perfusion in temporal and parietal areas [47]; modulating brainstem activities acting the descending pain pathways [48]; interacting, in a way still not well known, probably on the arousal network that presides over the cortical integration of the control of affective emotional expressions [8, 49].

On the other hand, dental ULFTENS, which can be considered in its own right as transcutaneous trigeminal stimulation, has been shown to be able to modulate the autonomic response (HRV) during a social stress test [13], and has demonstrated an effect of the pupillary light response differently in healthy and TMD subjects [50]. Dental ULFTENS, unlike other trigeminal transcutaneous electrical stimulation techniques, is delivered at a frequency of 0.66 hz (Ultra Low), also called Acupuncture-like. Part of its central effects on cardiovascular activity on the PAG-RVLM circuit (Periaqueductal gray-Rostroventrolater medulla circuit) [51, 52] and the vagal baroreflex circuit of the nucleus tractus solitarius [53] could be related to this mode of delivery. On the other hand, collaterals are sent from the PAG-RVLM circuit both to the locus coeruleus and to the caudal part of the nucleus of the solitary tract [54], largely justifying the functional relationships activated by ULFTENS between PAG-RVLM- Nago-LC nuclei.

The mechanism of action of generic Low frequency Tens on the endorphinic circuit associated with PAG-RVLM and serotonergic circuit of Rafe was elucidated by the group of Sluka (2001 & 2009) [55, 56]. Particularly low frequency Tens (4 hz), due to the prevalent action on mu opioid receptors, would act more centrally than high frequency Tens (100–120 hz) [57]. In addition, low-frequency (4 hz) electrical stimulation at the paravertebral level (T1-L2) induces a significant increase on HRV parameters (HF, LF, LF/HF) with an increase in HF and decrease in LF in subjects with essential hypertension [58]. Confirming previously described data for the "parasympathetic" effect of low-frequency (4 hz) tens on HRV [59].

There is, to our knowledge, no neuroanatomical or neurophysiological work expressly concerning the use of transcutaneous trigeminal electrical stimulation with Ultra Low frequencies. The interpretation for such a modality of trigeminal electrical stimulation remains, therefore, doubtful although it can be reasonably assumed that the mechanism of action is as described for Low Frequency Tens.

Transcutaneous electrical stimulation of the vagus is able to increase HRV in a manner dependent on the stimulation site at the level of the outer ear and the electrical mode of stimulation itself [60]. Some HRV parameters such as RMSSD and HF are significantly changed during auricular vagus stimulation [61]. Some recent data indicate that auricular stimulation of the vagus does not result in significant changes in HRV or other biomarkers [62–64], this, however, could be attributed to the significant differences between pulse delivery modes and the variety of physical characteristics of stimulation (frequency, amplitude for example). To our knowledge, however, no work has ever tested the stimulation frequency of 0.66 hz of dental ULF Tens, consequently it is difficult to make a comparison, even indirectly, between the results of work involving auricular vagus stimulation and ours.

Electrical stimulation of the dorsal surface of the tongue was first used to replace lost senses such as vision [65] and led to the idea that tongue sensory could be an effective tool to study and work through neuroplasticity of the central nervous system [15, 66]. In a general sense, the authors believe that the main effect is determined by the peculiarity of the trigeminal sensory innervation of the dorsal anterior mucosal surface of the tongue and the connections that the trigeminal nuclei have with the arousal pathways.

Sensory electrical stimulation of the dorsal anterior mucosa of the tongue has been used under the name cranial nerve noninvasive neuromodulation (CN-NINM) for various conditions such as balance disorders [67, 68] and gait disturbances in subjects with multiple sclerosis [69], to increase mobility in subjects with incomplete traumatic spinal cord injury [14], to improve The symptoms of subjects with mild to moderate traumatic brain injury [70], for tinnitus [71]. It has been suggested that its efficacy is due to the activation of the trigeminal nuclei at the pontine level, primarily, and the complex network they run to the vestibular nuclei, superior colliculus, various cerebellar and anterior cingulate cortex areas [72].

In mucosal sensory stimulation of the tongue, also referred to as Trans Lingual Neurostimulation (TLNS), the frequency of stimulation (Low, single pulse at 1.28 hz and High, triplets of pulses at 5 ms intervals (i.e., 200 Hz) every 20 ms (50 Hz)) has been tested, showing that HF appears to be more effective in increasing the alpha and theta activity of the high density EEG (electroencephalogram) of the resting brain in healthy subjects [73]. Neuromodulation through translingual neurostimulation (TLNS) has been shown to initiate long-lasting processes of neuronal reorganization observable through enhanced cognitive performance in healthy subjects [74].

In conclusion, our work, which should be considered a pilot study, showed that 6-minute sensory amplitude ULFTENS dental stimulation of the dorsal and anterior mucosal surface of the tongue with a low-cost and simple instrument induces a change of HRV in some of the parameters studied (DET and REC). This results in increased variability, increased complexity and a partial alteration in the activity of the autonomic nervous system. This result, probably, is determined by the activation and modulation of the areas of neuronal exchange between the trigeminal system and the brainstem system in charge of controlling cardiac activity including the vagal system and the system deputed to control arousal. The effect is maintained in the six minutes following stimulation indicating a plastic change in the circuits involved. This type of stimulation could be useful clinically in those subjects who show dysregulation of the autonomic system [75] and, in particular, who exhibit the alterations in HRV, features statistically associated with TMDs [76].

A limitation of our work is the type of study group selection. We chose healthy young women from the L’Aquila University student group. It might be possible that the stress undergone by college students could somehow affect the results of stimulation. Nevertheless, the control group is from the same selection and, therefore, should have no significant differences from the stimulated group of subjects. In fact, we had no significant differences in the baseline parameters of HRV. Overall, therefore, it is possible to consider that the effect on HRV parameters is not related to differences in selection or type of subjects.

A second limitation might be due to the selection of people belonging only to the female gender who, in fact, may be susceptible to hormonal factors that could influence the behavior of the autonomic system. We have tried to overcome this problem by avoiding the examination during the menstrual cycle phase, however, we have not excluded the ovulation period. Therefore, we cannot totally exclude the possibility of such influences on our results. On the other hand, randomization should have distributed the effect randomly and, therefore, reduced the impact on results. The choice of female gender only seemed referable, at this early stage of the study, because the percentage of subjects with TMD is higher with a ratio of 3 to 1 than the male gender.

In this pilot study, we do not have a comparison with placebo or sham tens. This can be considered a limitation of our work. It is a limitation that needs to be addressed, and we have adequate work planned to address this problem.

Another problem arises from the fact that throughout the trial the study subjects had the pacifier in their mouths. Thus, we cannot say whether part of the results are due to having the pacifier in their mouths as such, and therefore we cannot rule out a " pacifier " effect. Nevertheless, the data from the baseline condition (T0) involving the presence of the pacifier in the mouth are not statistically different from those of the control group making us assume that baseline in the first 6 minutes the pacifier effect is negligible, however, we cannot exclude that as time goes on the presence of the pacifier may not be influential. Future work should evaluate the effect on HRV of pacifier presence without stimulation.
